# Naturally Occurring tRNAs With Non-canonical Structures

**DOI:** 10.3389/fmicb.2020.596914

**Published:** 2020-10-21

**Authors:** Natalie Krahn, Jonathan T. Fischer, Dieter Söll

**Affiliations:** ^1^Department of Molecular Biophysics and Biochemistry, Yale University, New Haven, CT, United States; ^2^Department of Chemistry, Yale University, New Haven, CT, United States

**Keywords:** tRNA, non-canonical, genetic code expansion, identity elements, translation, selenocysteine, pyrrolysine, mitochondria

## Abstract

Transfer RNA (tRNA) is the central molecule in genetically encoded protein synthesis. Most tRNA species were found to be very similar in structure: the well-known cloverleaf secondary structure and L-shaped tertiary structure. Furthermore, the length of the acceptor arm, T-arm, and anticodon arm were found to be closely conserved. Later research discovered naturally occurring, active tRNAs that did not fit the established ‘canonical’ tRNA structure. This review discusses the non-canonical structures of some well-characterized natural tRNA species and describes how these structures relate to their role in translation. Additionally, we highlight some newly discovered tRNAs in which the structure–function relationship is not yet fully understood.

## Introduction

**FIGURE 1:**
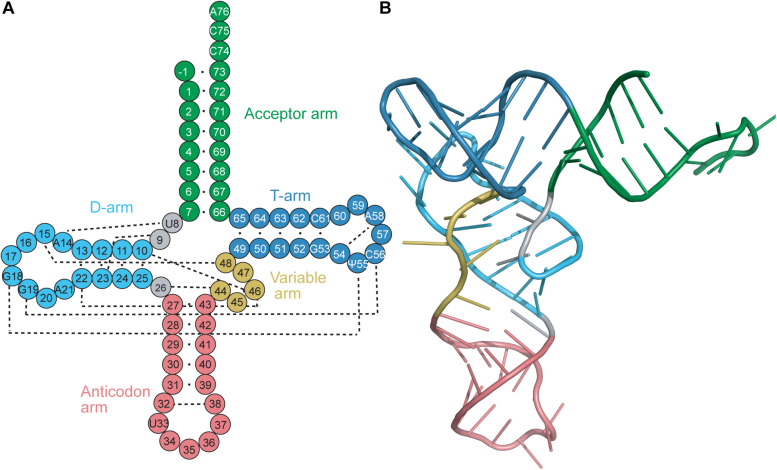
**(A)** Secondary and **(B)** tertiary representation of a tRNA molecule colored based on specific regions: acceptor arm (green), T-arm (dark blue), D-arm (light blue), variable arm (yellow), anticodon arm (pink). The cloverleaf (secondary structure) model includes the standard tRNA numbering. Dashed lines correspond to the tertiary interactions which form the L-shape observed with tRNAs.

Transfer RNAs (tRNAs) range in length between 70 and 100 nucleotides. tRNAs are acylated with the cognate amino acid by their cognate aminoacyl-tRNA synthetase (aaRS), and the resulting aminoacyl-tRNAs are substrates for ribosomal protein synthesis. tRNAs were determined early on to have a highly conserved cloverleaf secondary structure (Figure 1A) (Holley et al., 1965) and an L-shaped tertiary structure (Figure 1B) (Cramer et al., 1969). The cloverleaf secondary structure is formed from Watson–Crick base pairs (bp) which create helical stems typically ending in unpaired bases to form loops. These arms (stem and loop) include the acceptor arm, D-arm, anticodon arm, TΨC arm (T-arm), and a variable arm. Of these features, the acceptor arm, anticodon arm and T-arm are highly conserved in size, while the D-arm and variable arm can differ.

The stems of the acceptor arm and T-arm are found to have 7 and 5 bp, respectively, to give the canonical 7/5 configuration. Occasionally, a base-pair mismatch is found in the acceptor stem, which does not disrupt the conformation of the helix. Furthermore, the acceptor arm has four 3′-terminal residues which are not base paired: the discriminator base (Crothers et al., 1972) and the CCA tail. The 3′-terminal adenosine is in the form of a slightly activated ester to bind to the amino acid. The 3′-strand of the acceptor stem is directly connected to the T-stem, while the 5′-strand is connected to the D-stem by two unpaired bases. The length of the D-stem varies amongst tRNAs; most of them have between three and 5 bp leading up to the D-loop. The anticodon stem is also highly conserved in length, being found to consistently have 5 bp before ending with the anticodon loop. The anticodon loop has 7 nucleotides, with the three residues in the center of the anticodon loop (anticodon) participating in mainly Watson–Crick (but also sometimes non-Watson–Crick or wobble base) interactions with the codon of the mRNA. Finally, the variable loop is the least conserved amongst all tRNAs (Sigler, 1975). tRNAs are classified into two groups based on the size of their variable loops. Most tRNAs fall into class I and have four or five nucleotides in the variable loop, while class II tRNAs including tRNA^*Ser*^, tRNA^*Leu*^, and tRNA^*Tyr*^ have long variable loops consisting of 10 or more nucleotides (Sprinzl et al., 1998).

The conserved L-shape of tRNA is facilitated by base stacking and tertiary interactions between conserved or semi-conserved nucleotides. One arm of the L is formed from stacking of the acceptor stem with the T-stem, while the other arm is formed from stacking of the D-stem and anticodon stem. The conserved and semi-conserved nucleotides which form the tertiary interactions are found in the D- and T-loops; specifically G18 with Ψ55 and G19 with C56 (Figure 1A) (Hou, 1993). Additional tertiary interactions are found throughout the tRNA, including interactions between the D-stem and variable loop, the connecting base U8 and A14 in the D-loop, and stabilizing interactions within the T-loop and anticodon loop. Furthermore, stabilization often occurs at the level of the codon:anticodon interaction via tRNA modifications typically found at position 34, the wobble base in the anticodon, or position 37 which is just prior to the anticodon (Lyons et al., 2018). The conserved tRNA structure and sequences are crucial for functionality of the tRNA, including interaction with modifying enzymes (i.e., CCA-adding enzyme) and positioning in the ribosome (Lorenz et al., 2017).

Although tRNA structures are highly conserved, they do contain distinguishing elements which allow recognition by their cognate aaRS. These distinguishing elements, referred to as identity elements, are the only residues required for recognition by that aaRS. Common identity elements include the discriminator base and the anticodon; however, they are not limited to those regions. Identity elements of tRNAs have been extensively reviewed (Giegé et al., 1998) and therefore will not be discussed in detail here.

Instead, we focus on tRNAs with structures that deviate from canonical tRNAs. The nature of the proper secondary structure model of tRNAs was widely discussed (Hubert et al., 1998). Diverse experimental strategies (chemical and enzymatic RNA probing, phylogenetic analyses, and finally structural studies) showed the presence of natural tRNAs which lack the canonical 7/5 structure. As genomic studies expanded, many more tRNA genes with unique features were discovered. Some of these were poorly annotated due to the presence of unusual recognition elements, an anticodon sequence that disagreed with the other identity elements of the tRNA, or an irregular secondary structure.

## tRNA^*Sec*^

**FIGURE 2:**
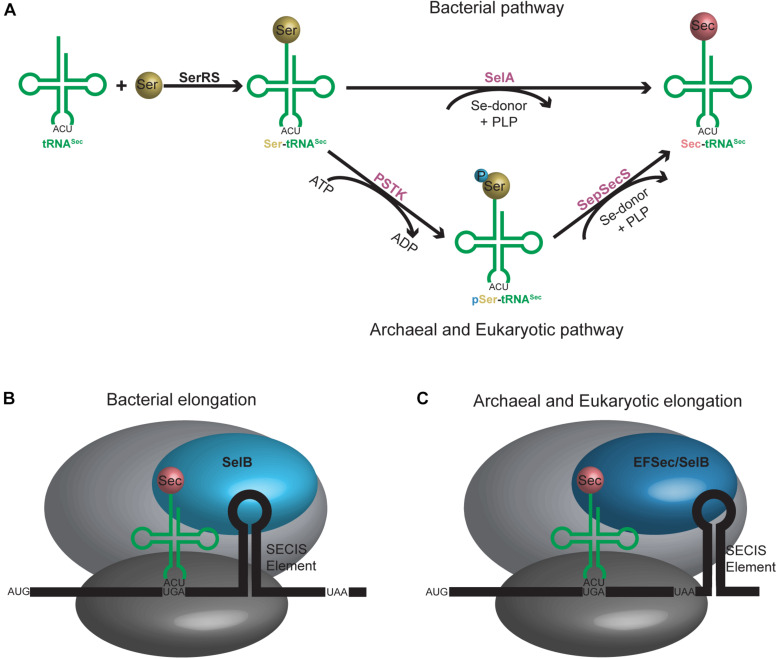
**(A)** Aminoacylation pathway for tRNA^*Sec*^ is different in bacteria compared to archaea and eukaryotes. An additional step is required in the latter, resulting in an intermediate tRNA^*Sec*^ containing a phosphoserine moiety. **(B)** Bacterial and **(C)** archaeal and eukaryotic elongation pathways for tRNA^*Sec*^. A selenocysteine insertion sequence (SECIS) element is required in the mRNA sequence which forms a hairpin in the 3′ translated region for bacteria or 3′ untranslated region for archaea and eukaryotes. A unique elongation factor [SelB (sometimes referred to as EFSec)] is required in all systems.

Discovered as the 21^*st*^ amino acid in 1976, incorporation of selenocysteine (Sec) into proteins occurs naturally in all domains of life (Cone et al., 1976). Unlike the translational mechanism of inserting the first 20 identified amino acids into proteins, incorporation of Sec into selenoproteins is more nuanced and involves additional steps. First, a specialized tRNA^*Sec*^ initially becomes aminoacylated with serine (Ser) by seryl-tRNA synthetase (SerRS) to form Ser-tRNA^*Sec*^. The serine hydroxyl group is then substituted with selenium to form Sec. In bacteria, this occurs in a single step with the enzyme selenocysteine synthase (SelA), while in archaea and eukaryotes, it is a two-step process involving first phosphorylation of the Ser with phosphoseryl-tRNA^*Sec*^ kinase (PSTK) followed by its replacement with selenium by *O*-phosphoseryl-tRNA^*Sec*^:Sec synthase (SepSecS) (Figure 2A). The fully aminoacylated Sec-tRNA^*Sec*^ product is then transported to the ribosome and incorporated into the nascent peptide at a UGA codon via a specialized elongation factor, SelB (also referred to as eEFSec in eukaryotes). SelB distinguishes UGA codons for Sec incorporation over UGA stop codons through recognition of a hairpin in the mRNA [**Sec I**nsertion **S**equence (SECIS) element] (Figures 2B,C) (reviewed in Serrao et al., 2018).

This rather complicated translational process is distinct from the translation pathway of the other 20 canonical amino acids. Therefore, it does not come as a surprise that tRNA^*Sec*^ does not conform to the structure of a canonical tRNA. From secondary structure predictions, it was evident that bacterial and archaeal tRNA^*Sec*^ both have a 13 bp acceptor domain (acceptor stem and T-stem) with an 8/5 and 9/4 structure, respectively (Schön et al., 1989; Sturchler et al., 1993; Hubert et al., 1998). However, this was not so clear for eukaryotes. The information obtained through modeling was torn between tRNA^*Sec*^ adopting a 9/4 structure like the archaeal tRNA^*Sec*^ or the canonical 7/5 structure observed in all other eukaryotic tRNAs known at the time (Ioudovitch and Steinberg, 1998; Steinberg et al., 1998). Without three-dimensional data to gather information from, researchers turned to phylogenetics to settle the debate. With eukaryotic and archaeal translational machineries being very similar to one another, it suggested that their tRNAs would have similar structures.

**FIGURE 3:**
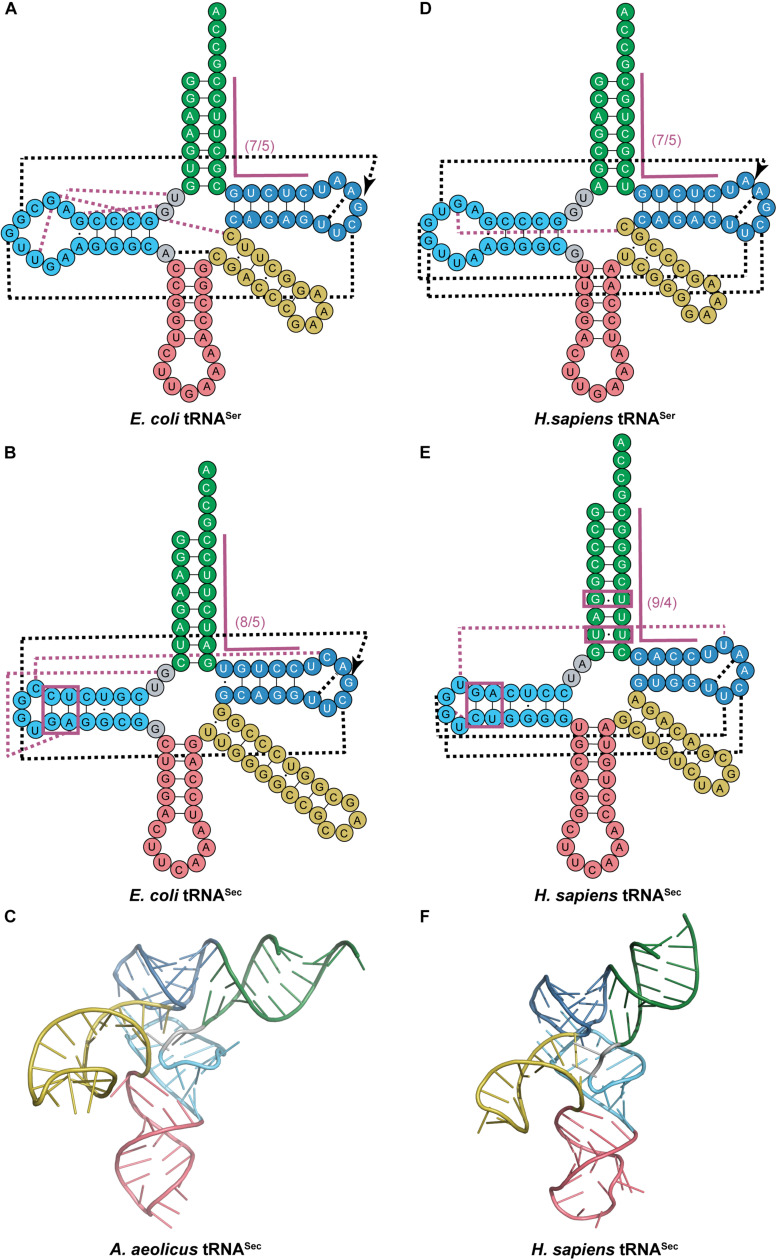
Comparing cloverleaf structures of tRNA^*Ser*^ with tRNA^*Sec*^ from bacterial and eukaryotic species with specific focus on the tertiary structure of tRNA^*Sec*^. Cloverleaf structures of **(A)** *E. coli* tRNA^*Ser*^ and **(B)** *E. coli* tRNA^*Sec*^ highlight differences in the acceptor domain and D-arm as well as different tertiary interactions. **(C)** Bacterial *A. aeolicus* tRNA^*Sec*^ (PDB ID: 3W3S; Itoh et al., 2013) forms the canonical L-shaped tertiary structure. A similar comparision is shown of **(D)** *H. sapiens* tRNA^*Ser*^ and **(E)** *H. sapiens* tRNA^*Sec*^. **(F)** The tertiary structure of *H. sapiens* tRNA^*Sec*^ (PDB ID: 3A3A; Itoh et al., 2009) also shows a similar L-shape. The tRNA structure elements are colored accordingly: acceptor arm (green), T-arm (dark blue), D-arm (light blue), variable arm (yellow), and anticodon arm (pink). Tertiary interactions are represented by dashed lines with black lines being conserved interactions between the two tRNAs while magenta lines are unique to that tRNA. Magenta boxes highlight important regions of tRNA^*Sec*^ for interaction with aminoacylation and elongation machinery.

This provided the necessary evidence for eukaryotic tRNA^*Sec*^ to be accepted as having a 9/4 structure even before the structural data was able to confirm it over a decade later (Hubert et al., 1998; Itoh et al., 2009; Palioura et al., 2009). The 9/4 structure of eukaryotic tRNA^*Sec*^ would imply that the 8^*th*^ residue would be base paired in the acceptor stem and unable to participate in binding to SerRS, a common interaction in canonical pairs of aaRSs and tRNAs. This is in contrast to what is found in *E. coli* tRNA^*Sec*^ with G8 forming a novel tertiary interaction with A21 and U14 (Sturchler et al., 1993). However, even with the proposed 9/4 arrangement, the overall tRNA secondary structure still has non-paired residues in positions 8 and 9 which in theory could participate in the aforementioned interactions. Crystal data show that this is not the case and there is instead an open cavity in the tertiary core with positions 8 and 9 not participating in any tertiary interactions. Instead, a different unique base triple is found in eukaryotic tRNA^*Sec*^ with U20 forming an interaction with the commonly found G19:C56 pair (Itoh et al., 2009). In addition to the canonical tertiary interactions between the D- and T-loops, a novel interaction was found between C16 in the D-loop and C59 in the T-loop for *E. coli* which is similar to the U16:U59 interaction in eukaryotic tRNA^*Sec*^. Moreover, the tertiary interactions found between the variable arm and D-arm in canonical tRNAs are absent in tRNA^*Sec*^ (Figure 3). Although this was predicted to create a different orientation of the variable arm with respect to the overall L-shape of the tRNA through biochemical studies (Baron et al., 1993), structures indicate that indeed the variable arm of tRNA^*Ser*^ and tRNA^*Sec*^ are in an almost identical orientation with respect to the T-arm (Itoh et al., 2009).

The unique structure of tRNA^*Sec*^, mainly the 13 bp acceptor domain structure and long variable arm, are essential for its function in translation (Mizutani and Goto, 2000). First, tRNA^*Sec*^ must be recognized by SerRS, which suggests it must have the same identity elements as tRNA^*Ser*^. Since SerRS only recognizes class II tRNAs, it follows that tRNA^*Sec*^ is also a class II tRNA (Schatz et al., 1991; Heckl et al., 1998). More specifically, it is the long variable arm and G73 discriminator base which SerRS recognizes for serylation of both tRNA^*Ser*^ and tRNA^*Sec*^ (Breitschopf and Gross, 1994). The mechanism of how the long variable arm serves as an identity element remains to be elucidated. It is hypothesized that the orientation of the arm is more important than the actual sequence based off of the low conservation over different tRNA^*Ser*^ sequences (Wu and Gross, 1993), but further studies suggest that there might be some sequence identity in the variable arm of tRNA^*Sec*^ (Ohama et al., 1994). This coincides with the evidence that SerRS preferentially binds 12 bp acceptor domain tRNAs. To get efficient serylation of tRNA^*Sec*^, which has a 13 bp acceptor domain, specific residues in the variable arm (and in the D-arm for eukaryotes) work together to promote synthetase binding and thus serylation (Ohama et al., 1994; Amberg et al., 1996; Fu et al., 2018).

Following serylation by SerRS, bacterial Ser-tRNA^*Sec*^ must then be recognized by SelA for conversion to Sec-tRNA^*Sec*^. One of the striking features that distinguishes tRNA^*Sec*^ from tRNA^*Ser*^ other than the acceptor domain, is the secondary structure of the D-arm. The D-stem in tRNA^*Sec*^ has 6 bp with a 4 base D-loop while tRNA^*Ser*^ has a 4 bp D-stem and 8–11 base D-loop (Figures 3A,B). Kinetic data initially suggested that the unique D-arm of bacterial tRNA^*Sec*^ was the important feature for SelA discrimination against tRNA^*Ser.*^ This was later confirmed by crystal structures to show that the N-terminal domain (NTD) of SelA is responsible for interacting with the D-arm of tRNA^*Sec*^ (Figure 3C) (Itoh et al., 2013). Further studies found that the interaction of SelA is not sequence-based but rather structural. The presence of the 5^*th*^ and 6^*th*^ bp in the D-arm of tRNA^*Sec*^ (regardless of sequence) had a positive impact on its activity (Ishii et al., 2013).

In eukaryotes, it is PSTK that must recognize Ser-tRNA^*Sec*^ for phosphorylation while at the same time excluding Ser-tRNA^*Ser*^. Similar to bacteria, the length and secondary structure of the D-arm differs between eukaryotic tRNA^*Sec*^ and tRNA^*Ser*^. The D-stem in tRNA^*Sec*^ has 6 bp with a 4-base D-loop, while tRNA^*Ser*^ has a 4 bp D-stem and 8-base D-loop (Figures 3D,E). Studies show that by simply adding 2 bp into the D-stem of tRNA^*Ser*^, it becomes a substrate for PSTK. Alternatively, by increasing the 4-base D-loop of tRNA^*Sec*^ to 8 bases, phosphorylation is decreased (Wu and Gross, 1994). These results suggest that the D-arm is a major identity element for PSTK recognition. It was also discovered that a minor contributor for successful phosphorylation is the T-stem length (tRNA^*Sec*^ has a 4 bp T-stem and tRNA^*Ser*^ has 5 bp). This was observed by the slight decrease in phosphorylation found by increasing the length of the T-stem in tRNA^*Sec*^ (Wu and Gross, 1994). These findings were confirmed by the complex crystal structure. Crystal contacts were found between PSTK and the D-arm and T-stem of tRNA^*Sec*^ (Figure 3F) (Chiba et al., 2010). In contrast to eukaryotic tRNA^*Sec*^, the archaeal D-stem is 7 bp and not considered a major identity element for PSTK recognition (reduction to 5 bp caused only a minor decrease in phosphorylation). Instead, the 13 bp acceptor domain binds to the NTD of PSTK as the major contributor (Sherrer et al., 2011) while the minor contributor was formed through the D-stem binding the C-terminal domain (CTD) of PSTK (Sherrer et al., 2008).

After phosphorylation by PSTK, archaeal and eukaryotic tRNA^*Sec*^ must also be recognized by SepSecS to form Sec-tRNA^*Sec*^ for incorporation into the polypeptide chain. As with the other enzymes involved in Sec incorporation, SepSecS distinguishes tRNA^*Sec*^ from the canonical tRNAs by the 13 bp acceptor domain (Figure 3E) (Amberg et al., 1996). The role of the 13 bp acceptor domain was determined to play a role in stabilization of tRNA^*Sec*^ for interaction with SepSecS. The co-crystal structure revealed that the main interaction of tRNA^*Sec*^ with SepSecS is through its acceptor domain, where it approaches the tRNA from the variable arm side and not through recognition of the D-arm (Palioura et al., 2009).

The final step in translation of tRNA^*Sec*^ is elongation. The unique structure of tRNA^*Sec*^ is also required for recognition by SelB and rejection from the traditional elongation factors (EF-Tu in bacteria and EF-1α in eukaryotes) used for canonical tRNAs. In bacteria, EF-Tu was found to bind with 100-fold weaker affinity to Ser-tRNA^*Sec*^ than Ser-tRNA^*Ser*^. This was found to be a result of the longer acceptor stem in tRNA^*Sec*^ (8 bp compared to 7 bp). This long acceptor stem is also the major structural determinant for SelB binding and the feature which distinguishes tRNA^*Sec*^ from the canonical tRNAs (Forster et al., 1990; Baron and Böck, 1991; Sturchler-Pierrat et al., 1995). With eukaryotes and archaea, the structural determinant for elongation is more specific. Phylogenetic considerations showed conservation of U6:U67 and a non-Watson–Crick base pair at 5a:67b in the acceptor stem of vertebrates, *Drosophila* and *Caenorhabditis elegans*. In vertebrates and *C. elegans* the sequence is a conserved wobble base pair G5a:U67b while in *Drosophila* it is replaced with A5a:G67b (highlighted in Figure 3E). Through randomization of both regions of the acceptor stem, it was found that the 5a:67b non-Watson–Crick interaction was imperative for function and that the U6:U67 pair was dispensable. The 5a:67b pair is believed to provoke structural modification of the phosphodiester backbone of the RNA helix for interaction with SelB (Mizutani et al., 1998b).

From the above evidence, the unique structure of tRNA^*Sec*^ is warranted by the specific interactions it encounters compared to canonical tRNAs. Interestingly, although multiple enzymes interact with tRNA^*Sec*^, none of them bind to the anticodon arm. Therefore, it follows that although tRNA^*Sec*^ was initially found to have a UCA anticodon and that majority of species conform to this, there are quite a few tRNA^*Sec*^ species with sense anticodons (Mukai et al., 2016). Through ongoing research in the field of Sec incorporation, a genomic search for tRNA^*Sec*^ in other species revealed two other tRNA variants: *selC*^∗^ tRNA^*Cys*^ and allo-tRNA (Mukai et al., 2017).

## tRNA^*Sec*^-Like Structures

In a metagenomic search for additional tRNA^*Sec*^ species, some tRNAs were found that are structurally similar to, but do not function as tRNA^*Sec*^. Further investigation into these unique structures led to the classification of two tRNA species: s*elC*^∗^ tRNA^*Cys*^ and allo-tRNA. Both tRNA groups contain the same distinctive tRNA^*Sec*^ structural features; a longer variable arm, acceptor stem, and anticodon stem compared to canonical tRNAs (Mukai et al., 2017).

### selC^∗^ tRNA^*Cys*^

**FIGURE 4:**
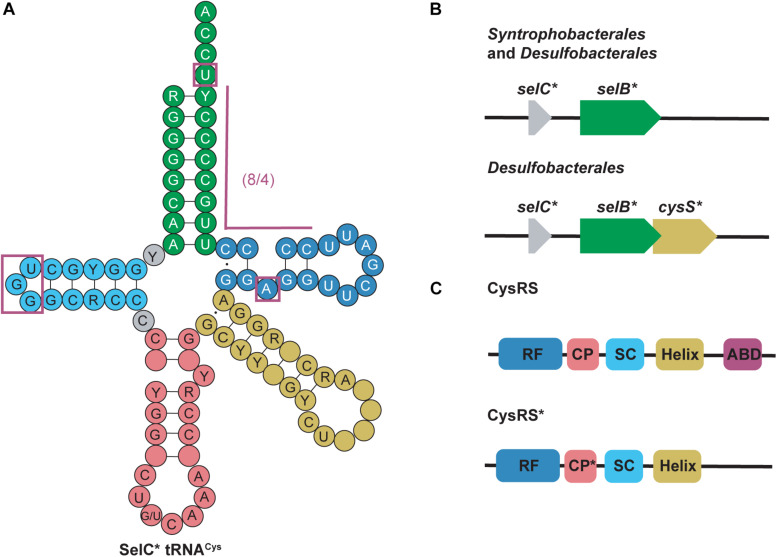
**(A)** Cloverleaf structure of SelC^∗^ tRNA^*Cys*^ highlights its unique structure compared with canonical tRNAs. Magenta boxes emphasize these specific regions. R and Y denote A/G and U/C, respectively and empty circles represent no conservation in sequence. **(B)** Genomic structure of selC^∗^ reveals an additional elongation factor (*selB*^∗^) in *Syntrophobacterales* and *Desulfobacterales* while an additional aaRS (*cysS*^∗^) is present in only *Desulfobacterales*. **(C)** *cysS*^∗^ (which codes for CysRS^∗^) is an aaRS that has a mutated connective polypeptide (CP^∗^) domain and the anticodon binding domain (ABD) is absent. The Rossman fold (RF) and stem-contact (SC) fold are consistent between CysRS and CysRS^∗^

*selC*^∗^ tRNA^*Cys*^ are found in anaerobic bacteria from the phyla *Firmicutes*, *Thermodesulfobacteria*, *Nitrospirae*, and *Proteobacteria*. s*elC*^∗^ tRNAs were named after the *selC* gene which encodes tRNA^*Sec*^ in *E. coli* (Mukai et al., 2017). *selC*^∗^ tRNA^*Cys*^ isoacceptors have similar structure to tRNA^*Sec*^ but contain identity elements of tRNA^*Cys*^ (notably the U73 discriminator base and cysteine (GCA) anticodon) (Pallanck et al., 1992; Komatsoulis and Abelson, 1993). Although the GCA anticodon is a strong identity element for CysRS recognition, some of the *selC*^∗^ tRNA^*Cys*^ were found to have an opal (UCA) anticodon instead. Further evidence showed that some CysRS variants can cysteinylate tRNA^*Cys*^_*UCA*_, therefore including this group of tRNAs in this category (Turanov et al., 2009). The most striking feature of s*elC*^∗^ tRNA^*Cys*^ is their modified 8/4 structure. In the 4 bp T-stem an unpaired adenosine produces a bulge at position 51a. This is similar to what is found in the structure of minor bacterial (8/4) tRNA^*Sec*^ species (Figure 4A) (Mukai et al., 2017).

Further genomic analysis revealed that in two δ-proteobacterial subgroups, *Syntrophobacterales* and *Desulfobacterales*, a second copy of *selB* (*selB*^∗^) was found downstream of the *selC*^∗^ genes. From this, it was hypothesized that *selC*^∗^ tRNA^*Cys*^ is recognized by SelB^∗^ in a similar way as the 8/4 tRNA^*Sec*^ is recognized by SelB. Moreover, in *Desulfobacterales*, *selC*^∗^ tRNA^*Cys*^ was found to contain an A1:U72 pair and an opal (UCA) anticodon. As previously mentioned, some CysRS variants (encoded by *cysS*) would be able to recognize the opal anticodon, however, they would be unable to recognize the A1:U72 pair. Therefore in these species a second copy of CysRS was found to be encoded downstream of *selB*^∗^ (*cysS*^∗^) (Figure 4B) (Mukai et al., 2017). CysRS^∗^ lacks an anticodon binding domain, which allows for recognition of *selC*^∗^ tRNA^*Cys*^ with an opal anticodon. Recognition of A1:U72 is possible due to mutations in the CP1 domain (Figure 4C) (Liu et al., 2012). These discoveries suggest that CysRS^∗^ specifically evolved to recognize and aminoacylate *selC*^∗^ tRNA^*Cys*^. *In vivo* analysis confirmed that CysRS^∗^ can aminoacylate *selC*^∗^ tRNA^*Cys*^ through recognition of the 8 bp acceptor stem and the unique A51a bulge, characteristic of *selC*^∗^ tRNA^*Cys*^, was found to be dispensable (Mukai et al., 2017).

### Allo-tRNA

**FIGURE 5:**
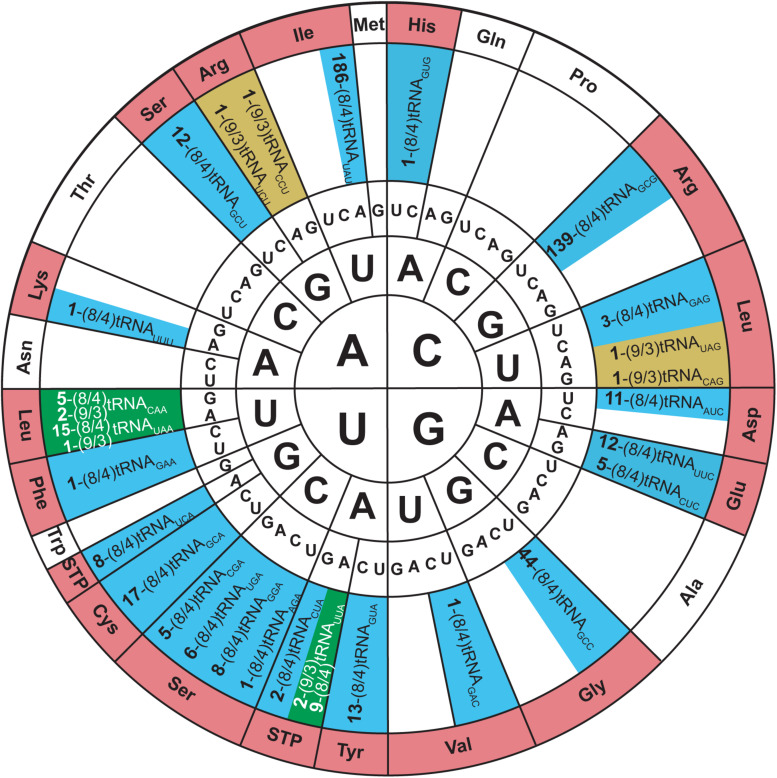
Genetic code wheel highlights the codons and subsequent amino acids which can be incorporated by allo-tRNAs of 9/3 structure (yellow), 8/4 structure (blue) or both (green) (adapted from Mukai et al., 2017). The number of allo-tRNAs found with the indicated anticodon (subscript) is shown as a bold number in front of the allo-tRNA name. Some allo-tRNAs with a specific anticodon are thought to be able to read through multiple codons.

Allo-tRNA genes belong to bacteria from *Clostridia*, *Proteobacteria*, and *Acidobacteria*. They encode a unique tRNA whose striking feature is their 12 bp acceptor domain, which is found in either an 8/4 or 9/3 conformation (Mukai et al., 2017). Based on the presence of identity elements for SerRS recognition, allo-tRNAs were suggested to be Ser isoacceptors. With the knowledge that SerRS can recognize not only 7/5 tRNA^*Ser*^ but also 8/5, 9/4, and 8/4 tRNA^*Sec*^, this was a reasonable hypothesis (Mizutani et al., 1998a). Moreover, most allo-tRNAs were found to have non-serine anticodons which SerRS does not recognize (Breitschopf and Gross, 1994). In fact, the anticodons are highly diverse and span 35 out of 64 codons. The most predominant anticodons for the 8/4 allo-tRNA species are UAU, GCG, and GUC which correspond to isoleucine (Ile), arginine (Arg), and aspartic acid (Asp), respectively. Conversely, anticodons UUC, GUC, CAC, and AAA corresponding to phenylalanine (Phe), valine (Val), histidine (His), and lysine (Lys) were only found once in the metagenomic data analyzed. Fewer 9/3 species were found and they contained anticodons which corresponded to Arg, leucine (Leu) and the ochre stop codon (UAA) (Figure 5) (Mukai et al., 2017).

*In vivo* studies began to examine the utility of these tRNAs in the bacterial translation system. Initially, two allo-tRNAs (an 8/4 and 9/3 structure) from *Silvibacterium bohemicum* were expressed in *E. coli* with position 2 of super-folder green fluorescent protein (sfGFP) mutated to Leu codons (CUC and UUA). Interestingly, Ser was efficiently incorporated into sfGFP as confirmed by fluorescence and mass spectrometry data. Other 8/4 and 9/3 allo-tRNAs were found to contain the major identity element for recognition by alanyl-tRNA synthetase (AlaRS), specifically the G3:U70 wobble base pair (Hou and Schimmel, 1988; Mcclain and Foss, 1988). Testing their capabilities *in vivo*, it was found that Ala and Ser were the main residues incorporated at an amber codon; however, insertion of other amino acids including Asn, Gln, Lys, Cys, Ile, and Glu were also detected. These studies showed that allo-tRNAs derived from other bacterial species could be efficiently used as a substrate in the *E. coli* translation system, and the nature of the incorporated amino acid is based on the allo-tRNA identity elements rather than on the anticodon present (Mukai et al., 2017).

**FIGURE 6:**
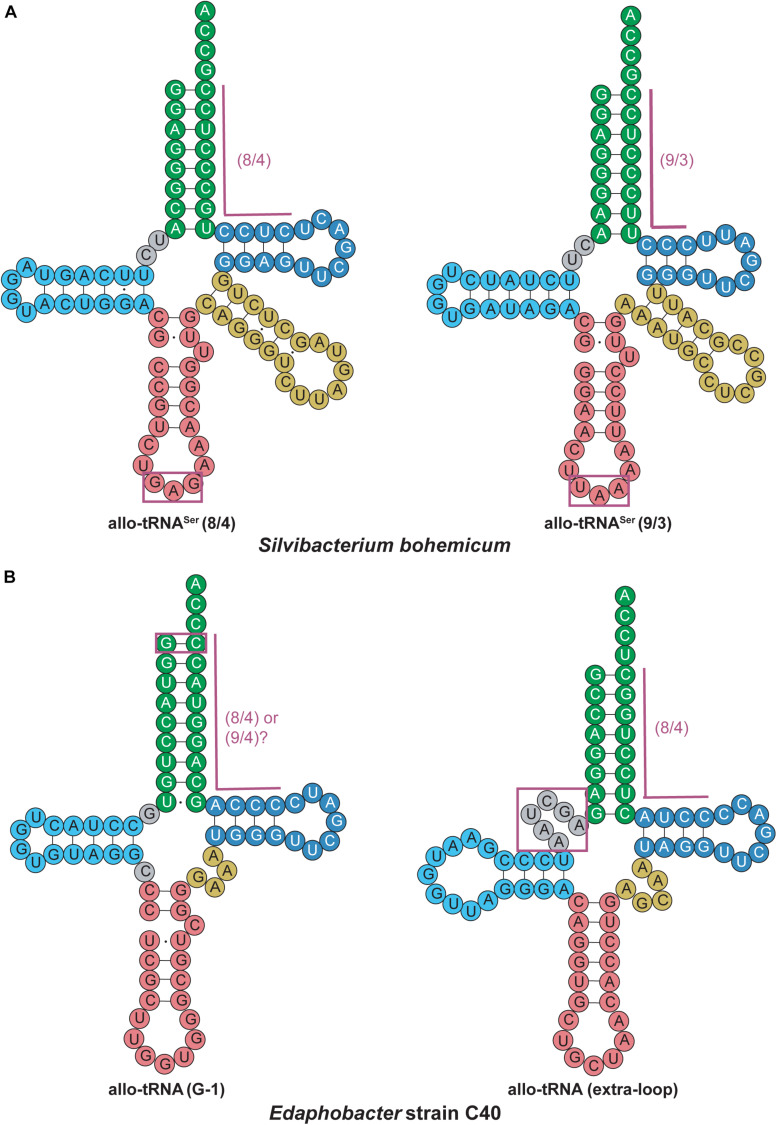
Allo-tRNA secondary structures for **(A)** *Silvibacterium bohemicum* and **(B)** *Edaphobacter* strain C40 have unique features from canonical tRNAs. **(A)** Both 8/4 and 9/3 structures are observed for allo-tRNA^*Ser*^ with anticodons (boxed in magenta) that do not correlate with decoding of the Ser amino acid. **(B)** Two completely different tRNA secondary structures are proposed with either a G at the –1 position to form an additional base pair (boxed in magenta) providing either a 8/4 or 9/4 structure and a tRNA structure with an additional fifth loop between the acceptor arm and D-arm (gray boxed in magenta).

An interesting discovery was found in the *Edaphobacter* strain C40. An allo-tRNA_*UAU*_ pseudogene with several base-pair disruptions was found overlapping with the open reading frame of a transposon-related protein. This allo-tRNA species was found to be the most abundant group among the allo-tRNA genes observed in the soil and sediment metagenomic sequences. Their cloverleaf structures were unlike allo-tRNA^*Ser*^, containing stem-destabilizing mutations as in the *Edaphobacter* strain C40 and possible five-stem-junction structures (Figure 6). Unlike the previously described allo-tRNAs, these were unable to be used for translation in *E. coli* and were hypothesized to be associated with transposable elements or toxin-antitoxin systems. Moreover, polycistrons of allo-tRNA-like sequences and other irregular tRNA sequences were discovered from forest and peat soil metatranscriptomic data. Many of these tRNAs were predicted to have an 8/4 structure, but with additional features were found, including an extra loop in between the acceptor stem and D-stem as well as a G-1 base. These allo-tRNAs could not be aminoacylated by *E. coli* aaRSs *in vitro*. However, this does not answer the question whether they are used for translation in their original hosts with an aaRS capable of recognizing these unique differences in the tRNA structure (Mukai et al., 2017).

## tRNA^*Pyl*^

Pyrrolysine (Pyl), the 22^*nd*^ proteinogenic amino acid, was discovered in the active site of methylamine methyltransferase in the archaeal methanogen *Methanosarcina barkeri* (Hao et al., 2002). Pyl is genetically encoded via an in-frame amber (UAG) codon, which is normally used as a stop codon to terminate protein synthesis. This is possible due to an amber suppressor tRNA found in certain archaeal and bacterial species, pyrrolysine tRNA (tRNA^*Pyl*^) (Srinivasan et al., 2002). tRNA^*Pyl*^ is aminoacylated by its cognate pyrrolysyl-tRNA synthetase (PylRS), a class II aaRS (Polycarpo et al., 2004). Unlike Sec, which requires a multi-step enzymatic process to be incorporated into a protein during translation (see section “tRNA^*Sec*^”), incorporation of Pyl utilizes the same translational machinery as canonical tRNAs (Théobald-Dietrich et al., 2004; Zhang et al., 2005; Longstaff et al., 2007). The PylRS-tRNA^*Pyl*^ pair has been studied extensively; it is frequently utilized as a tool for genetic code expansion due to its ability to charge a wide variety of non-canonical amino acids (ncAAs) as well as its orthogonality in both bacterial and eukaryotic hosts (Wan et al., 2014; Tharp et al., 2018).

**FIGURE 7:**
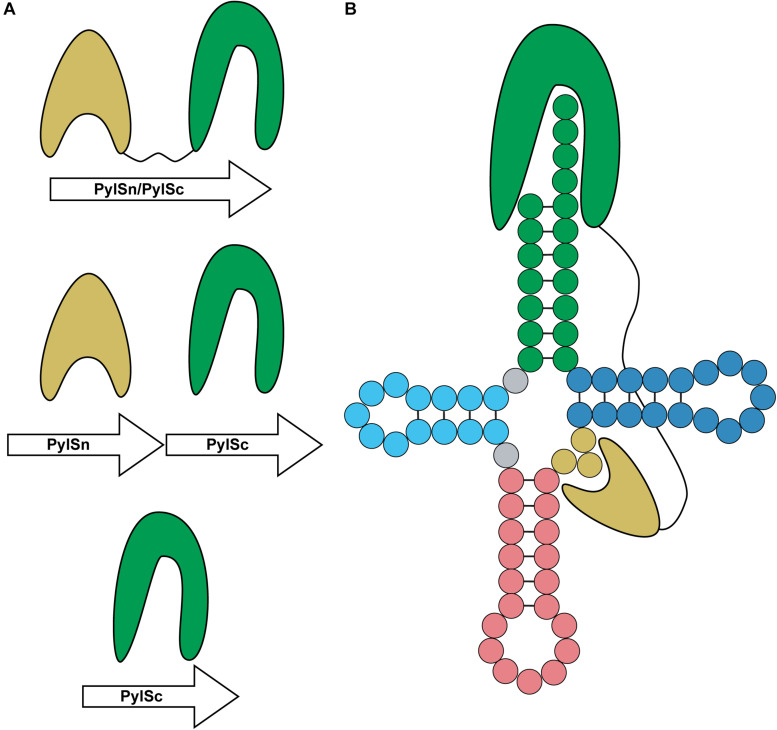
Domain organization and binding mode of PylRS. **(A)** PylRS is composed of two domains, an N-terminal domain (PylSn) and a catalytic domain (PylSc). PylRS is either composed of a fusion of these two domains, two standalone proteins, or as a lone PylSc. **(B)** PylSc interacts with the acceptor stem and catalyzes the aminoacylation of tRNA^*Pyl*^. PylSn forms a tight interaction with the variable arm.

PylRS is typically composed of two domains: a CTD catalytic domain (PylSc) and an NTD (PylSn) (Figure 7A) (Herring et al., 2007a). The organization of these domains varies between organisms. In species from the archaeal genus *Methanosarcina*, PylRS is encoded as a single protein featuring both an NTD and CTD connected with a linker (Herring et al., 2007a). On the other hand, Pyl-utilizing bacteria such as *Desulfitobacterium hafniense* encode two individual proteins, PylSc and PylSn, for each domain (Nozawa et al., 2009). Finally, seventh-order methanogens such as *Methanomethylophilus alvus* encode a protein homologous to PylSc, but no homolog of PylSn exists in these archaea (Borrel et al., 2014). In general, PylSc is responsible for catalyzing the aminoacylation of tRNA^*Pyl*^, while PylSn forms additional contacts with the tRNA (Figure 7B) (Herring et al., 2007a; Nozawa et al., 2009; Suzuki et al., 2017). Regardless of the domain structure of the enzyme, tRNA^*Pyl*^ structure and its interaction with PylRS varies from canonical tRNAs, and at the same time vary from one another.

Unlike previously mentioned tRNAs, most tRNA^*Pyl*^ species characterized to date have the canonical 7/5 tRNA structure, which allows translation with the same machinery as canonical tRNAs. Crystal structures as well as structure mapping and melting curve assays show that tRNA^*Pyl*^ adopts a tertiary conformation similar to the canonical L-shape (Théobald-Dietrich et al., 2004; Nozawa et al., 2009). The distinguishing features of tRNA^*Pyl*^ are the three-nucleotide variable arm, an elongated anticodon stem (from 5 to 6 bp), and a CUA anticodon. More specifically, the universal tRNA^*Pyl*^ identity elements are the discriminator base G73, and the first bp in the acceptor stem G1:C72 (Ambrogelly et al., 2007; Herring et al., 2007b). However, since these are also identity elements of many other tRNAs (Giegé et al., 1998; Giegé and Frugier, 2000–2013), additional identity elements are necessary for PylRS to distinguish tRNA^*Pyl*^ from the canonical tRNAs. These additional identity elements can differ for each PylRS-tRNA^*Pyl*^ pair and therefore will be explored in more detail below.

**FIGURE 8:**
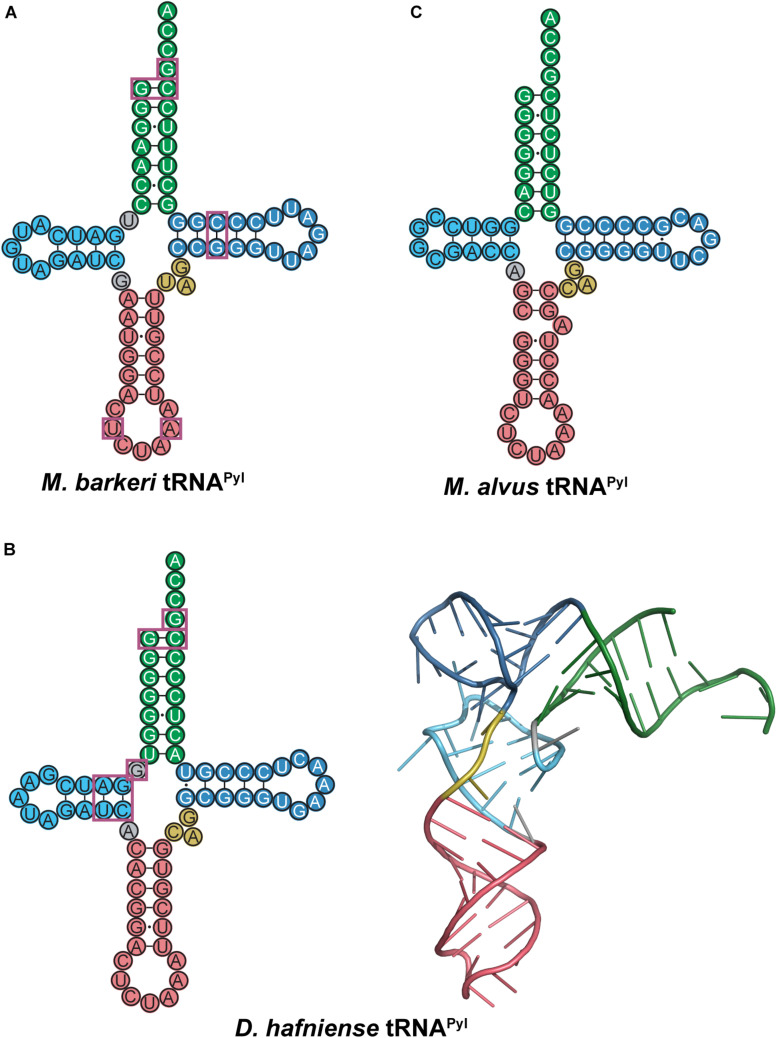
Cloverleaf structures of tRNA^*Pyl*^ from **(A)** *M. barkeri*, **(B)** *D. hafniense*, and **(C)** *M. alvus*. Identity elements for each tRNA^*Pyl*^ are highlighted by magenta boxes. The crystal structure of *D. hafniense* tRNA^*Pyl*^ (PDB ID: 2ZNI; Nozawa et al., 2009) is also shown in **(B)**.

The *M. barkeri* tRNA^*Pyl*^ (*Mb* tRNA^*Pyl*^) contains the above-mentioned features of tRNA^*Pyl*^ with a 6 bp anticodon stem (Figure 8A). However, it contains some additional features that differ from canonical tRNAs and distinguishes it from other tRNA^*Pyl*^ species. Canonical tRNAs contain two nucleotides between the acceptor stem and D-stem, while *Mb* tRNA^*Pyl*^ only has one. However, the connecting nucleotide is a U, consistent with the highly conserved U8 in canonical tRNAs. Furthermore, the D-loop is small, with only five nucleotides, and lacking the widely conserved G18, G19 sequence motif. Since the D- and T-loop are known to interact with each other, it follows that the T-loop is missing the corresponding U54, Ψ55, and C56 sequence. The absence of G19 and C56 (which forms a tertiary interaction in canonical tRNAs) indicates that an unusual interaction occurs between the D- and T-loops in *Mb* tRNA^*Pyl*^. Details on the identity elements of *Mb* tRNA^*Pyl*^ were elucidated by screening its amber suppression efficiency (Ambrogelly et al., 2007). This study revealed that the nucleotides adjacent to the anticodon U33 and A37, and the T-stem bp G51:C63 are identity elements. Mutation of these identity elements significantly decreased the binding of *Mb* tRNA^*Pyl*^ to *Mb* PylRS in addition to their suppression efficiency. Furthermore, transplanting these identity elements into bovine mitochondrial tRNA^*Ser*^ yielded an active chimeric tRNA that could be aminoacylated by *Mb* PylRS both *in vitro* and *in vivo*.

The crystal structure of *M. mazei* tRNA^*Pyl*^ (*Mm* tRNA^*Pyl*^) in complex with *M. mazei* PylSn (NTD) revealed the importance of the small, three-nucleotide variable arm. A tight interaction is formed between *Mm* PylSn and the variable arm of tRNA^*Pyl*^ (Suzuki et al., 2017). As previously mentioned, the small variable arm is a unique feature of tRNA^*Pyl*^, as the variable arms of canonical tRNAs typically have 4–5 nucleotides for class I tRNAs, or greater than 10 nucleotides in the case of class II tRNAs (Sprinzl et al., 1998). Therefore, PylSn discriminates against canonical tRNAs based on the size of their variable arm. Addition of a fourth nucleotide to the variable arm of *Mb* tRNA^*Pyl*^ significantly decreases its suppression efficiency, providing further evidence that the interaction between PylRS and the variable arm is critical for aminoacylation (Ambrogelly et al., 2007).

Although there are several differences in nucleotide sequences, the secondary structure of *Mb* tRNA^*Pyl*^ is quite similar to the homologous tRNA from *D. hafniense*. Like *Mb* tRNA^*Pyl*^, *Dh* tRNA^*Pyl*^ has an elongated anticodon stem, shortened D-loop, small variable arm, and lacks the conserved nucleotide sequences G18, G19, and TΨC (Figure 8B). However, *Dh* tRNA^*Pyl*^ is unique from tRNA^*Pyl*^ from *methanosarciniae* in that the single nucleotide separating the acceptor and D-stem is G8 as opposed to U8. In canonical tRNAs, this position is widely conserved as U8, which stabilizes tertiary structure through base pairing with A14. Thus, the absence of U8 in *Dh* tRNA^*Pyl*^ abolishes the highly conserved U8:A14 bp (Herring et al., 2007b; Nozawa et al., 2009).

The crystal structure of the *D. hafniense* PylSc in complex with *Dh* tRNA^*Pyl*^ shows that the change of U8 to G8 allows an unusual interaction to occur between the D- and T-loop, wherein G13 interacts with C55 to stabilize the tertiary conformation of the tRNA (Nozawa et al., 2009). This also enables G8 to serve as an identity element for the interaction with PylSc, specifically through interaction with residues Arg140, Arg144, and Glu145 (Herring et al., 2007b; Nozawa et al., 2009). Despite these differences, *Dh* tRNA^*Pyl*^ folds into an L-shape similar to canonical tRNAs (Figure 8B), with a compact core that is accommodated by the PylSc active site.

Structural and biochemical data on the interaction between *Dh* tRNA^*Pyl*^ and *Dh* PylSc have revealed several tRNA identity elements (Figure 8B). In addition to the universal tRNA^*Pyl*^ identity elements, a direct interaction occurs between *Dh* PylSc and the D-stem base pairs G10:C25 and A11:U24, as well as the previously mentioned G8 (Herring et al., 2007b; Nozawa et al., 2009). Although *in vitro* aminoacylation assays indicate that the nucleotides flanking the anticodon U33 and A37 are identity elements for *Mb* PylRS (Ambrogelly et al., 2007), *Dh* PylSc and PylSn do not directly interact with these residues (Nozawa et al., 2009; Jiang and Krzycki, 2012). Furthermore, while the anticodon is normally a tRNA identity element, *Dh* PylSc is found not to interact with the anticodon, which is also the case for all other characterized PylRS-tRNA^*Pyl*^ pairs (Ambrogelly et al., 2007; Herring et al., 2007b; Nozawa et al., 2009). This desirable trait allowed for general codon reassignment, and thus opened the door for synthetic biologists to incorporate multiple ncAAs into a single protein using different PylRS-tRNA^*Pyl*^ pairs (Wan et al., 2010; Chin, 2017). Ultimately, *Dh* PylSc binds to tRNA^*Pyl*^ through contacts with the acceptor and D-stem, and has no direct contact with the anticodon stem, variable loop, or T-stem (Nozawa et al., 2009).

The PylRS-tRNA^*Pyl*^ pair in the seventh-order methanogen *M. alvus* has recently been explored as an additional tool for genetic code expansion with advantages over its previously studied counterparts (Meineke et al., 2018; Willis and Chin, 2018; Yamaguchi et al., 2018; Beránek et al., 2019; Dunkelmann et al., 2020; Seki et al., 2020). *Ma* tRNA^*Pyl*^ has many unusual features that distinguish it from canonical tRNAs as well as previously characterized tRNA^*Pyl*^ (Figure 8C). The anticodon stem of *Ma* tRNA^*Pyl*^ features 6 bp in the anticodon stem like other tRNA^*Pyl*^, but the stem is broken by an unpaired adenosine on the 3′ side of the stem. Other seventh order methanogens such as *Methanomassiliicoccus intestinalis* and *Methanomassiliicoccus lumenyensis* tRNA^*Pyl*^ feature larger breaks that form small loops within the anticodon stem (Borrel et al., 2014). Also, *Ma* tRNA^*Pyl*^ does not have a nucleotide separating the acceptor and D-stem of the tRNA. This differs considerably from canonical tRNAs as well as from tRNA^*Pyl*^ species previously mentioned. An additional difference of *M. alvus* tRNA^*Pyl*^ is the four nucleotide D-loop (instead of five observed in the other tRNA^*Pyl*^ discussed).

On the surface, the break in the base pairing of the anticodon stem as well as the lack of a connecting base between the acceptor and D-stem profile as potential identity elements for *Ma* tRNA^*Pyl*^. Interestingly, deletion of the unpaired nucleotide in the anticodon stem did not significantly alter the translation efficiency of *Ma* PylRS-tRNA^*Pyl*^ in a cell-free translation system (Yamaguchi et al., 2018). Insertion of a C or U between the acceptor and D-stem (position 8) moderately decreased translation, but inserting an A or G had no effect (Yamaguchi et al., 2018). This indicates that the absence of a base in this position may not be an identity element for *Ma* tRNA^*Pyl*^. Therefore, in this system, the functional role, if any exists, of these unique features of *Ma* tRNA^*Pyl*^ is unclear.

Unlike *M. barkeri* and *D. hafniense*, *M. alvus* does not encode a protein homologous to PylSn, either as a standalone protein or a fusion to PylSc. PylSn binds tightly to the variable loop of tRNA^*Pyl*^ (Suzuki et al., 2017) and is essential for *in vivo* aminoacylation of *Dh* tRNA^*Pyl*^ (Herring et al., 2007a). However, *Ma* PylRS is highly active toward its cognate tRNA^*Pyl*^ even though it does not feature PylSn. Despite significant structural differences between *Ma* and *Mm* tRNA^*Pyl*^, *Ma* tRNA^*Pyl*^ can serve as a substrate for both PylRS enzymes (Yamaguchi et al., 2018). However, lengthening the variable arm of *Ma* tRNA^*Pyl*^ prevents aminoacylation by *Mm* PylRS, due to steric constraints between PylSn and the enlarged variable arm as discussed earlier (Suzuki et al., 2017). Since *Ma* PylRS does not have a PylSn to interact with the variable arm, it still readily aminoacylates the tRNA despite the larger variable arm (Willis and Chin, 2018).

## Mitochondrial tRNAs

Mitochondria are responsible for energy production in eukaryotic cells. As a semi-autonomous organelle descendent from bacteria, mitochondria have their own genome. Mitochondrial genomes not only encode proteins essential for energy production, but also encode parts of the translation machinery, including mitochondrial tRNAs (mt-tRNAs) (Gray et al., 1999). The number of mt-tRNA genes encoded in the mitochondrion varies between organisms. In most cases, mitochondria import additional, nuclear-encoded tRNA and proteins that are required for translation (Alfonzo and Söll, 2009; Dudek et al., 2013; Salinas-Giegé et al., 2015). In addition to mt-tRNAs, mitochondrial translation occurs via a specialized translation machinery, including mitoribosomes and mitochondrial initiation and elongation factors (Salinas-Giegé et al., 2015; D’ Souza and Minczuk, 2018). Although canonical tRNAs require conserved structural elements for proper folding, many mt-tRNAs possess highly unusual secondary structures that deviate greatly from canonical tRNAs.

**FIGURE 9:**
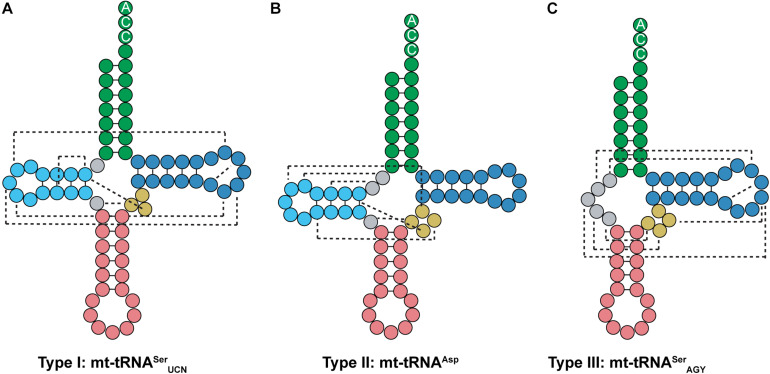
Mammalian mt-tRNA can be classified into three types. **(A)** Type I mt-tRNA, represented by mt-tRNA^*Ser*^_*UCN*_, shares similarities with canonical tRNAs, featuring the same conserved D- and T-loop interactions. **(B)** Type II mt-tRNA, represented by mt-tRNA^*Asp*^, is the most commonly found mt-tRNA. **(C)** Type III mt-tRNA, represented by mt-tRNA^*Ser*^_*AGY*_, do not have a D-arm. Instead, the connecting region between the acceptor and anticodon stem interacts with the variable and T-loop to promote folding.

Most tRNAs found in organisms are type 0 tRNAs, which have a conserved cloverleaf structure and fold into a tertiary L-shape due to interactions between the D- and T-loops. On the other hand, mt-tRNAs can be classified into three types based off of their secondary structure (Watanabe, 2010; Suzuki et al., 2011). Type I mt-tRNAs have an atypical anticodon stem. This includes mt-tRNA^*Ser*^_*UCN*_, which has 6 bp in the anticodon stem instead of the typical 5 bp (Figure 9A). Mammalian mt-tRNA^*Ser*^_*UCN*_ has many similarities with *Mb* tRNA^*Pyl*^ that are not seen in most characterized tRNAs. Both tRNA structures have only a single nucleotide separating the acceptor and D-stem, have smaller than normal D-loops, elongated anticodon stems, and variable arms consisting of only three nucleotides. However, unlike *Mb* tRNA^*Pyl*^, mt-tRNA^*Ser*^_*UCN*_ features the G18, G19, and TΨC sequences in its D- and T-loops (Figures 7A, 9A). Furthermore, type I mt-tRNAs have the L-shaped tertiary structure which resembles that of canonical tRNA (Watanabe et al., 1994a; Hayashi et al., 1998; Mustoe et al., 2015). The most common mt-tRNAs, type II mt-tRNAs lack interaction between the D- and T-loops (Figure 9B). In these mt-tRNAs, the canonical G18, G19, and TΨC sequence motifs in the D- and T-loop, respectively, are not conserved. Instead, interactions occur between the D-loop and the variable stem to stabilize the mt-tRNA tertiary structure (Wakita et al., 1994; Messmer et al., 2009; Watanabe, 2010). Finally, type III mt-tRNAs lack a D-stem; they are the only mammalian mt-tRNAs without the canonical cloverleaf structure. An example of a type III mt-tRNA is mt-tRNA^*Ser*^_*AGY*_ (Figure 9C). Despite lacking a D-stem, this mt-tRNA is functional *in vitro* and adopts a conformation that is suitable for the ribosome (Hanada et al., 2001; Frazer-Abel and Hagerman, 2008).

The interaction between mt-tRNAs and mt-aaRSs is not well-understood, as there is limited structural information available on the binding of mt-tRNAs to their cognate aaRSs. However, identity elements have been established for mammalian mt-tRNA^*Tyr*^ (Bonnefond et al., 2005, 2007), mt-tRNA^*Leu*^ (Sohm et al., 2003, 2004), mt-tRNA^*Ser*^ (AGY and UCN) (Shimada et al., 2001), mt-tRNA^*Ala*^ (Lovato et al., 2001), and mt-tRNA^*Asp*^ (Fender et al., 2006; Neuenfeldt et al., 2013) species. Interestingly, mammalian mt-aaRSs appear to have evolved relaxed specificity for their cognate tRNAs. Specifically, bovine mt-aaRSs have been shown to acylate the corresponding *E. coli* tRNAs, while the *E. coli* aaRSs cannot acylate the equivalent mt-tRNA (Kumazawa et al., 1991; Watanabe et al., 1994a). Mt-SerRS is even more promiscuous, as it serylates several *E. coli* tRNAs as well as mt-tRNA^*Gln*^ (Shimada et al., 2001). Mt-tRNA^*Gln*^ is an orphan tRNA; in addition to being a substrate for mt-SerRS, mt-tRNA^*Gln*^ is also mischarged by mt-GluRS to Glu-tRNA^*Gln*^, which is subsequently transamidated to Gln-tRNA^*Gln*^ (Nagao et al., 2009). In canonical aaRS-tRNA pairs, the first bp is a common identity element. However, in the more promiscuous human mt-TyrRS it was found not to recognize the first bp of mt-tRNA^*Tyr*^ (Bonnefond et al., 2005, 2007). Taken together, these findings indicate that in mammals, mt-aaRSs do not strongly discriminate against non-cognate tRNAs. This apparent lack of specificity may be attributed to the high substrate diversity of mt-tRNAs, or possibly a lack of evolutionary pressure due to the smaller pool of mt-tRNAs present in the cell.

**FIGURE 10:**
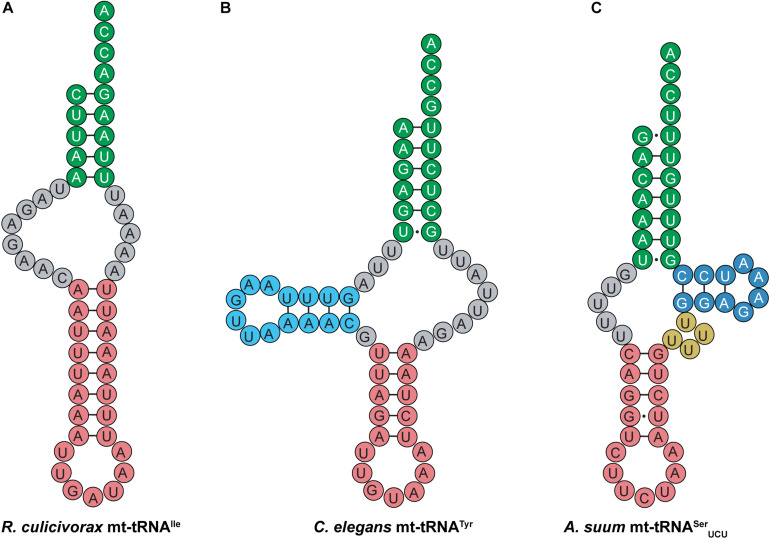
Nematode mt-tRNAs have diverse and highly unusual secondary structures. Examples of these abnormal mt-tRNA structures are shown here. **(A)** *R. culicivorax* mt-tRNA^*Ile*^ has no D- or T-arm. **(B)** *C. elegans* mt-tRNA^*Tyr*^ has a D-arm, but no T-arm. **(C)** *A. suum* mt-tRNA^*Ser*^_*UCU*_ has a short T-arm and a variable loop, but no D-arm.

Like their mammalian counterparts, nematode mt-tRNAs have unusual structural features that are distinct from canonical tRNAs. Nematodes encode short mt-tRNAs with diverse cloverleaf structures. In addition to nematodes, highly truncated mt-tRNAs have been found in the genomes of mites and arachnids, where short tRNAs missing both the D- and T-arms have been identified (Figure 10A) (Klimov and Oconnor, 2009; Jühling et al., 2012; Palopoli et al., 2014; Wende et al., 2014; Salinas-Giegé et al., 2015; Juhling et al., 2018). Despite greatly deviating from the canonical tRNA cloverleaf structure, evidence suggests that mt-tRNAs lacking one or both sidearms can still interact with tRNA processing enzymes such as CCA-adding enzyme, and are aminoacylated by their cognate synthetases (Wolfson et al., 1999; Tomari et al., 2002; Wende et al., 2014). Most nematode mt-tRNAs lack the entire T-arm (Figure 10B), except mt-tRNA^*Ser*^, which have a short T-arm consisting of only 10–13 nucleotides (Wolstenholme et al., 1987; Okimoto and Wolstenholme, 1990; Watanabe et al., 1994b). For instance, *Ascaris suum* mt-tRNA^*Ser*^_*UCU*_ has a short, 10-nucleotide T-arm, and completely lacks a D-arm (Figure 10C) (Ohtsuki et al., 2002). The short T-arm in *A. suum* tRNA^*Ser*^_*UCU*_ as well as the connector region which replaces the D-arm, confer flexibility to the mt-tRNA. This flexibility allows the mt-tRNA to adopt a less rigid tertiary structure than the canonical L-shape, enabling the distance between the 3′ end of the tRNA and the anticodon to properly adjust to fit into the ribosome (Ohtsuki et al., 2002). Similar findings of flexible tertiary structure have been reported for mammalian mt-tRNA^*Ser*^_*AGY*_ (Figure 9C), which also lacks a D-arm (Steinberg and Cedergren, 1994; Frazer-Abel and Hagerman, 2008). In addition to these observations, recent structural data also indicate that *R. culicivorax* mt-tRNA^*Ile*^ (Figure 10A) folds into a stable, boomerang-shaped tertiary structure (Juhling et al., 2018). Further, the 3D structure reveals that the distance between the anticodon and 3′ end of *R. culicivorax* mt-tRNA^*Ile*^ is comparable to that of canonical, cytosolic tRNA^*Phe*^ from *Saccharomyces cerevisiae* (Juhling et al., 2018). Thus, evidence suggests that the D- and T-arms are not required for tRNA to fold into a tertiary conformation suitable for enzymatic activity, and the flexibility of these truncated mt-tRNAs helps to achieve functionality.

In addition to the flexible tertiary structure discussed above, post-transcriptional modifications appear to play an important role in stabilizing mt-tRNAs. Many unmodified mt-tRNAs will not fold properly, but proper modification allows folding and interaction with tRNA processing enzymes to occur (Lorenz et al., 2017). For instance, 1-methyl adenosine at position 9 (m^1^A9) is found in many mt-tRNA species, including those lacking one or both sidearms, and this modification is important for proper cloverleaf folding to occur (Helm et al., 1999; Sakurai et al., 2005; Lorenz et al., 2017; Juhling et al., 2018). Nematode mt-tRNA lacking the D-arm possess m^1^A9 as well as several pseudouridine in the acceptor and anticodon stem (Sakurai et al., 2005; Lorenz et al., 2017; Juhling et al., 2018). In these mt-tRNA, m^1^A9 facilitates aminoacylation and interaction with EF-Tu (Sakurai et al., 2005), while pseudouridine likely supports tRNA stability (Lorenz et al., 2017). Ultimately, post-translational modification appears to play an important role in facilitating mt-tRNA activity and stability, including the truncated mt-tRNAs that lack a canonical cloverleaf structure.

Deviations from the standard genetic code have been reported in the mitochondria of green plant algae from the phylum Chlorophyta (Noutahi et al., 2019). Similar to the pyrrolysine incorporation system in archaea and bacteria, the stop codons UAG and UGA are reassigned to sense codons in some Chlorophyta. In several of these species, UAG is reassigned to Ala or Leu, and UGA is reassigned to Trp (Fučíková et al., 2014). These mt-tRNAs with CUA or UCA anticodons, feature identity elements of tRNA^*Ala*^, tRNA^*Leu*^ or tRNA^*Trp*^ species, thus allowing for stop codon suppression and elongation with the corresponding amino acid.

Recent evidence suggests that in addition to stop codon reassignment, sense codons may also be reassigned in green algae. AGG, which is normally an Arg codon, appears to be reassigned in Sphaeropleales (Noutahi et al., 2019). In these green algae, mt-tRNAs with a CCU anticodon do not share any structural or sequence similarities with canonical tRNA^*Arg*^. Analysis of the mt-tRNA_*CCU*_ secondary structures reveals that many of these mt-tRNAs instead share identity elements with Chlorophyta mt-tRNA^*Ala*^_*UGC*_, including the invariant G3:U70 pair and the discriminator base A73.

Sense codon reassignment has also been observed in *S. cerevisiae* mitochondria as well as that of *Ashbya gossypii*, a relative of yeast (Alfonzo and Söll, 2009; Su et al., 2011; Ling et al., 2014, 2015). In *S. cerevisiae* mitochondria, CUN codons, which normally decode Leu, are reassigned to Thr. This reassignment is facilitated by an unusual mt-tRNA^*Thr*^_*UAG*_ that features an enlarged 8-nt anticodon loop and a UAG anticodon. *S. cerevisiae* lacks mt-tRNA^*Leu*^_*UAG*_, thus allowing complete reassignment of the CUN codon from Leu to Thr. Interestingly, phylogenetic and mutational analyses of yeast mt-tRNAs indicate that mt-tRNA^*Thr*^_*UAG*_ evolved from mt-tRNA^*His*^_*GUG*_ as opposed to mt-tRNA^*Leu*^_*UAG*_ or mt-tRNA^*Thr*^_*UGU*_ (Su et al., 2011). In *A. gossypii*, the codons CUU and CUA are reassigned to decode Ala. Like the Chlorophyta mt-tRNA_*CCU*_ described above, *A. gossypii* mt-tRNA^*Ala*^_*UAG*_ features the strictly conserved Ala identity element G3:U70. This bp is critical for recognition of mt-tRNA^*Ala*^_*UAG*_ by AlaRS, with a G3A mutation abolishing aminoacylation (Ling et al., 2014). The observation that codon reassignment occurs in mitochondria across kingdoms underscores the dynamic nature of the mitochondrial genome.

## Outlook

This evidence shows that not all tRNAs have the canonical 7/5 structure that was originally portrayed. The unique structures found in these non-canonical tRNAs appear to be a result of their necessary function and the enzymes that they interact with. In some cases (tRNA^*Sec*^), these details are well-understood while in others (allo-tRNAs) it is a bit more speculative. Despite deviating from the canonical structure, majority of the tRNAs presented in this review have been found to be functional in translation. While tRNA^*Sec*^ has specialized translational requirements for Sec to be incorporated into proteins, tRNA^*Pyl*^ utilizes the same translational machinery as canonical tRNAs (Théobald-Dietrich et al., 2004; Zhang et al., 2005; Longstaff et al., 2007). In mitochondria, highly unusual mt-tRNAs with diverse structures are used along with specialized mitochondrial translation machinery to translate proteins encoded by the mitochondrial genome (Gray et al., 1999). Taken together, these observations clearly indicate that the canonical tRNA structure is not a prerequisite for translation, and it is evident that although canonical tRNAs are in the 7/5 structure, their translation systems can accommodate diverse structures including 8/4, 8/5, 9/3, and 9/4 structures.

The translational machinery has evolved to accept a wide variety of tRNA structures for efficient translation of proteins in the desired host. A significant amount of effort has been put forth to expand the genetic code, pushing the boundaries of what functionality can be incorporated into proteins. To that end, engineered aaRS-tRNA pairs have been utilized to incorporate numerous diverse ncAAs into proteins both *in vitro* and *in vivo*. In many cases, the host’s translation machinery readily accepts foreign and modified tRNAs featuring diverse structures and charged with a ncAA (Chin, 2017). This plasticity may indicate a lack of evolutionary pressure to discriminate against unknown or unusual tRNAs that are rarely, if ever, encountered by the host cell. Thus, it is plausible that the unusual structures of specialized or non-canonical tRNAs such as the ones described in this review are made possible by a lack of evolutionary pressure to maintain the canonical structure. An alternative possibility is that many of these non-canonical tRNAs originated from an ancient, more diverse genetic code, and because of their specialized and infrequent usage, they were never pressured to evolve into a canonical tRNA structure. In either case, if deviations from the canonical tRNA structure are well-tolerated by the aminoacyl-synthetase and translation machinery, mutations or structural changes to the tRNA can potentially occur without consequence and lead to polymorphisms over time. This can be seen in mitochondria. Highly variable mt-tRNAs are well-known to be susceptible to mutations, and while mt-tRNA mutations to critical nucleotides can cause diseases, neutral or slightly deleterious polymorphisms frequently occur and are inconsequential (Lynch, 1996; Wittenhagen and Kelley, 2003; Yarham et al., 2010). Ultimately, despite their many differences from the canonical tRNA structure, non-canonical tRNAs are readily utilized in translation and enable the cell to produce proteins that are, in many cases, essential for survival (Longstaff et al., 2007).

## Author Contributions

NK and JF wrote the manuscript. DS edited the manuscript. All authors contributed to the article and approved the submitted version.

## Conflict of Interest

The authors declare that the research was conducted in the absence of any commercial or financial relationships that could be construed as a potential conflict of interest.
